# Early Tensile Loading in Nonsurgically Treated Achilles Tendon Ruptures Leads to a Larger Tendon Callus and a Lower Elastic Modulus: A Randomized Controlled Trial

**DOI:** 10.1177/03635465221117780

**Published:** 2022-08-25

**Authors:** Zlatica Rendek, Leo Bon Beckman, Thorsten Schepull, Ida Dånmark, Per Aspenberg, Jörg Schilcher, Pernilla Eliasson

**Affiliations:** † Author deceased; * Orthopedic Department, Linköping University Hospital, Linköping, Sweden; ‡ Department of Biomedical and Clinical Sciences, Faculty of Medicine and Health Science, Linköping University, Linköping, Sweden; Investigation performed at the Faculty of Medicine and Health Science, Linköping University, Linköping, Sweden

**Keywords:** Achilles tendon rupture, tendon healing, loading, rehabilitation, nonsurgical treatment

## Abstract

**Background:**

Early tensile loading improves material properties of healing Achilles tendon ruptures in animal models and in surgically treated human ruptures. However, the effect of such rehabilitation in patients who are nonsurgically treated remains unknown.

**Hypothesis:**

In nonsurgically treated Achilles tendon ruptures, early tensile loading would lead to higher elastic modulus 19 weeks after the injury compared with controls.

**Study Design:**

Randomized controlled trial; Level of evidence, 2.

**Methods:**

Between October 2015 and November 2018, a total of 40 nonsurgically treated patients with acute Achilles tendon rupture were randomized to an early tensile loading (loaded group) or control group. Tantalum bead markers were inserted percutaneously into the tendon stumps 2 weeks after the injury to allow high-precision measurements of callus deformation under mechanical testing. The loaded group used a training pedal twice daily to produce a gradual increase in tensile load during the following 5 weeks. Both groups were allowed full weightbearing in an ankle orthosis and unloaded range of motion exercises. Patients were followed clinically and via roentgen stereophotogrammetric analysis and computed tomography at 7, 19, and 52 weeks after the injury.

**Results:**

The mean ± standard deviation elastic modulus at 19 weeks was 95.6 ± 38.2 MPa in the loaded group and 108 ± 45.2 MPa in controls (*P* = .37). The elastic modulus increased in both groups, although it was lower in the loaded group at all time points. Tendon cross-sectional area increased from 7 weeks to 19 weeks, from 231 ± 99.5 to 388 ± 142 mm^2^ in the loaded group and from 188 ± 65.4 to 335 ± 87.2 mm^2^ in controls (*P* < .001 for the effect of time). Cross-sectional area for the loaded group versus controls at 52 weeks was 302 ± 62.4 mm^2^ versus 252 ± 49.2 mm^2^, respectively (*P* = .03). Gap elongation was 7.35 ± 13.9 mm in the loaded group versus 2.86 ± 5.52 mm in controls (*P* = .27).

**Conclusion:**

Early tensile loading in nonsurgically treated Achilles tendon ruptures did not lead to higher elastic modulus in the healing tendon but altered the structural properties of the tendon via an increased tendon thickness.

**Registration:**

NCT0280575 (ClinicalTrials.gov identifier).

Achilles tendon ruptures are common injuries, and the incidence is increasing.^14,24^ A variety of treatment options are available. Open surgical treatment with sutures is the first choice for many surgeons mainly because of a lower risk for reruptures.^27,39^ Other treatment alternatives include percutaneous suture fixation and various types of nonsurgical treatment.^27^ Traditional treatments, regardless of whether they are surgical or nonsurgical, entail several weeks of immobilization in a cast or orthosis.

To overcome longstanding functional impairment, a modern physical therapy approach has been implemented and includes early functional rehabilitation with early weightbearing while wearing an ankle orthosis and unloaded ankle joint motion exercises.^15,45^ This shift in rehabilitation, away from extensive periods of immobilization and unloading toward early loading and motion, has been suggested to reduce the overall rerupture rate and, in combination with the lack of surgery-associated risks, has made nonsurgical treatment more popular.^14,16,23,27,39^

Early functional rehabilitation can be applied without affecting the risk of tendon elongation after either surgical or nonsurgical treatment.^12,22,25,28,31^ Although joint mobility has been shown to be improved,^8,28^ neither unloaded ankle motion exercise nor weightbearing appears to improve the material properties of the tendon callus or reduce muscle atrophy.^4,12^ Animal studies have shown that early loading increases the tendon callus strength and that different load levels lead to differences in structural and material properties of the tendon.^1^ Small amounts of continuous loading (complete unloading vs moderate loading) mainly cause improved material properties of the callus tissue, whereas heavy loading results in a thicker callus but not necessarily better material properties. In surgically treated patients, early tensile loading twice daily, starting 2 weeks after the surgery, from 30 N with a gradual increase to 225 N, led to a higher elastic modulus at 19 weeks postoperatively.^36^ A higher elastic modulus at 19 weeks correlated with a superior heel-rise index at 1 year, thus indicating an improved healing process.^36^ Nevertheless, studies of this type of early tensile loading have not been conducted on nonsurgically treated Achilles tendon ruptures. Nonsurgically treated tendons might be more prone to elongation; however, another possibility is that nonsurgically treated tendons are more responsive to short loading stimulus and tissue deformation. The lack of suturing could result in tendon ends that are less stable and vascularity of the tissue that is not disturbed by a surgical procedure and strangulating sutures, similar to indirect bone healing.^33^

With the shift toward more frequent use of nonsurgical treatment, it is important to elucidate the effect of early tensile loading and to discriminate thresholds of loading that benefit or disturb the healing tendon. In this randomized controlled trial, our primary objective was to investigate whether early, gradually increased tensile loading in nonsurgically treated Achilles tendon ruptures would lead to faster recovery of tendon material properties measured via elastic modulus at 19 weeks after the injury. Secondary objectives were to investigate whether this rehabilitation regimen could prevent muscle atrophy and improve patient outcome measured via the Achilles tendon Total Rupture Score (ATRS).

Our main hypothesis was that short bouts of tensional loading, initiated 2 weeks after the injury, would lead to a tendon callus with higher elastic modulus. A secondary hypothesis was that early loading would increase patient satisfaction and reduce muscle atrophy.

## Methods

### Trial Design

**Figure 1.**
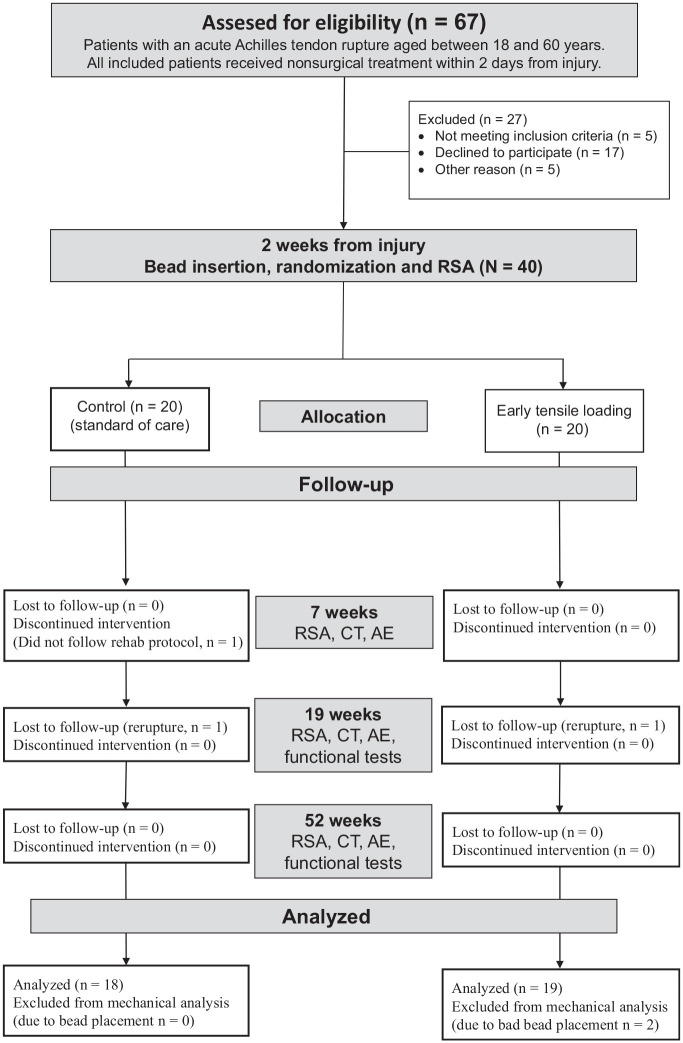
CONSORT (Consolidated Standards of Reporting Trials) diagram. AE, adverse event; CT, computed tomography; RSA, roentgen stereophotogrammetric analysis; Rehab, study rehabilitation protocol.

The study was performed as a single-center, randomized controlled trial with patients in 2 parallel groups, a loaded group (n = 20) and a control group (n = 20). All patients were recruited between October 2015 and November 2018 at Linköping University Hospital (Figure 1). Written informed consent was obtained from all participants. Ethical approval was obtained from the regional ethical review board in Linköping (No. 2014/69-31). The trial is registered with clinicaltrials.gov (NCT0280575).

### Inclusion and Exclusion Criteria

Patients who were between 18 and 60 years of age and had a complete midsubstance Achilles tendon rupture and a positive Thompson test were invited to participate in the study. All patients had received nonsurgical treatment within 2 days after the injury had occurred. This treatment entailed use of a removable ankle orthosis (foam walker boot; NovaWalk, JHInova AB) with the ankle in plantarflexion. Excluded were patients with a previous ipsilateral Achilles tendon injury, a rupture at the myotendinous junction or osteotendinous junction, systemic diseases affecting tendon healing (eg, diabetes mellitus or rheumatic diseases), and immunosuppressive treatment including systemic corticosteroids. Patients unable to understand the Swedish language were also excluded.

### Inclusion, Randomization, and Blinding

**Figure 2.**
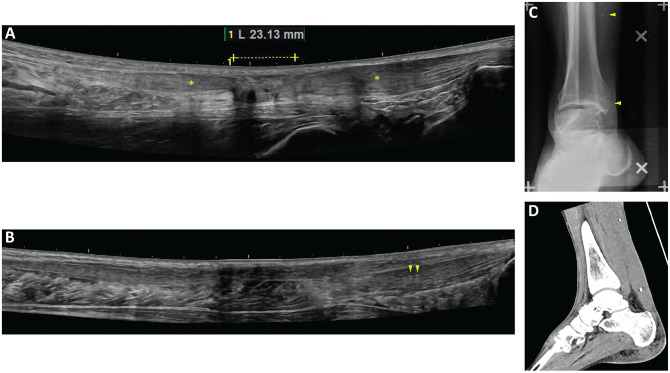
(A) Ultrasound image from an injured Achilles tendon indicating the injured hypoechogenic area (dotted line) and areas with a well-organized linear collagen structure where the proximal and distal beads are injected (asterisks). (B) Ultrasound image from a healed tendon injury (4 years after the injury). The distal beads are visible and indicated by arrowheads. Images from (C) roentgen stereophotogrammetric analysis (beads indicated by arrowheads) and (D) computed tomography (1 year after the injury).

Inclusion and randomization were done at approximately 14 days from injury, when tantalum beads were inserted. An ultrasound investigation was performed to localize the rupture site and validate the full-thickness tear. The rupture was defined as a defect in the tendon structure visualized as an hypoechogenic area on ultrasound examination (Figure 2). Adjacent to the hypoechogenic area in the proximal and distal directions, intact tendon tissue was identified as areas with a well-organized linear collagen structure in the fascicle of the tendon. These areas were marked using a pen on the skin, and the tissue depth was measured. With the patient under local anesthesia, using xylocaine with adrenaline, we injected 0.8-mm tantalum beads percutaneously via a 17-gauge Venflon needle, 2 beads in the proximal part and 2 beads in the distal part of the intact tendon tissue. Beads were inserted by Z.R., T.S., or P.A., and were used to assess the material properties and the gap elongation of the tendon using roentgen stereophotogrammetric analysis (RSA) (Figure 2), as previously described by Schepull and Aspenberg.^36^ Randomization was performed by using serially numbered, sealed envelopes by the principal investigator (P.E.). The envelope was opened after the beads were inserted. The randomization was conducted in blocks of 6 patients, with 1 random exchange of a pair of envelopes between the blocks, to prevent assumptions of group belonging. Patients were unblinded from the intervention, but outcome parameters were assessed by blinded assessors (Z.R., L.B.B.).

### Rehabilitation Interventions

**Figure 3.**
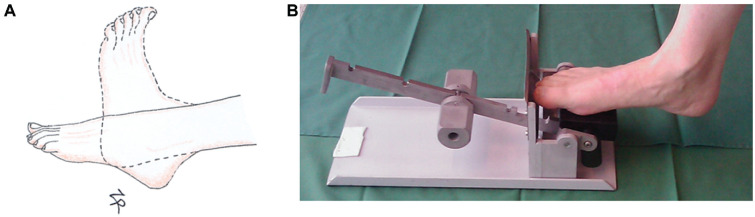
Training exercises. (A) Unloaded ankle motion exercises from the start of week 3 (both groups). The motion exercises consisted of 20 to 30 repetitions, performed 3 times per day, from maximal plantarflexion to the neutral position. (B) Training pedal used for early tensile loading, starting week 3. Initial pedal resistance of 30 N was increased each week to 75, 125, 175, and finally 225 N.

Both groups were treated with a removable ankle orthosis from the start. Two patients (1 patient in each group) received a cast with the ankle in plantarflexion for 2 and 4 days, respectively, and thereafter an orthosis. The patients were not allowed to remove the orthosis during the initial 2 weeks, and thereafter they were allowed to remove the orthosis only for personal hygiene and training exercises (Table 1). The standard of care at Linköping University Hospital includes active functional rehabilitation with weightbearing as tolerated on the orthosis using crutches from day 1. The orthosis initially contained 3 heel wedges. One wedge was removed at week 4, and another was removed at week 5; the last wedge was removed at week 6, resulting in a neutral position of the ankle joint. Unloaded ankle motion exercises without the orthosis were encouraged at week 3. The motion exercises consisted of 20 to 30 repetitions performed 3 times per day by moving the ankle joint from maximal plantarflexion to the neutral position in a sitting position (Figure 3). The number of repetitions was increased to 30 at the beginning of week 5. This active rehabilitation protocol was used in both study groups.

**Table 1:** Rehabilitation Program Guidelines

	Week After Injury												
	1	2	3	4	5	6	7	8	9	11	17	19	26
Ankle orthosis^*a*^	Yes	Yes	Yes	Yes	Yes	Yes	Yes						
No. of heel wedges	3	3	3	2	1	0	0						
No. of unloaded ankle exercises performed 3×			20	20	30	30	30						
Tensile loading 2 × 20			30 N	75 N	125 N	175 N	225 N						
Walking without orthosis^*b*^								Yes					
Sitting heel rise								Yes					
Exercise bicycle									Yes				
Standing double-leg heel rise										Yes			
Standing Single-leg heel rise											Yes		
Jogging												Yes	
Return to sports													Yes

a Full weightbearing as tolerated.

b Crutches were used until patients walked without a limp.

The loading group received exercise instructions at week 3 in addition to the active rehabilitation using an exercise pedal (Figure 3). After bead insertion, the patients received rehabilitation instructions from the study physical therapist and the study principal investigator. The patients were instructed to keep the foot in a slightly plantar flexed position and to resist dorsiflexion moment during the loading exercise. Patients were instructed to perform as many repetitions as possible twice daily, with a maximum of 20 repetitions allowed per session. The pedal had an initial resistance of 30 N, which was increased each week to 75, 125, 175, and finally 225 N. This regimen has previously been shown to result in higher elastic modulus at 19 weeks postoperatively in surgically treated Achilles tendon ruptures.^36^ The patients kept a diary of the number of repetitions performed at each session. All patients were followed up via telephone at 4 weeks, and the principal investigator remained accessible for study-related questions throughout follow-up.

Both groups removed the ankle orthosis after 7 weeks and were instructed to use crutches until they could walk without a limp (approximately 3-4 weeks later). All patients then started a regular rehabilitation program, which was the same for both groups. This program included home exercises together with regular visits to physical therapists who were unrelated to the study. All physical therapists followed regional guidelines for nonsurgically treated Achilles tendon ruptures, which included sitting heel rises starting week 8, double-leg standing heel rises starting approximately week 11, and single-leg standing heel rises starting approximately week 17; full activity including sports was allowed after approximately 6 months.

### Study Outcome

The primary outcome of this study was tendon elastic modulus at 19 weeks. Secondary outcomes were tendon elastic modulus at 7 and 52 weeks, tendon cross-sectional area, gap elongation (ie, the distance between the beads), creep, heel-rise index, patient-reported outcome according to the ATRS, ankle range of motion, and circumference of the calf. Exploratory variables were circumference of the ankle and visual analog scale (VAS) score for pain at 7 weeks.

### Computed Tomography

Computed tomography of the injured leg, between the proximal and distal tantalum beads, was done at 7, 19, and 52 weeks to calculate tendon cross-sectional area. The measurements were performed blinded from all other patient-related information by 1 author (L.B.B.) under the supervision of the principal investigator. The border of the Achilles tendon was manually outlined using the measurement toolbox of the Sectra IDS7 software (Version 19.1.8.3542; Sectra AB). Mean cross-sectional area was determined from 3 consecutive transversal cuts for further calculations. The computed tomography images were also used to exclude beads outside the tendon tissue. This appeared in 6 patients at 7 weeks. Of these patients, 4 patients had an additional bead inserted before the 19-week follow-up and could participate in mechanical testing at 19 and 52 weeks. These patients were excluded from the analysis of gap elongation over time.

### Roentgen Stereophotogrammetric Analysis

RSA was used to measure tendon strain at 7, 19, and 52 weeks after injury. RSA can be used to measure the distance between tantalum beads in 3 dimensions.^42^ During the procedure, the patients sat on an examination table with the foot in a custom-made frame, with 8° of plantarflexion as previously described.^2^ The frame contained a pedal under the forefoot allowing the application of various external loads: 10 kg at 7 weeks; 2.5 and 15 kg at 19 weeks; and 2.5, 22.5, and 35 kg at 52 weeks (Table 2). The pedal pivoted around an axis with an adjustable distance from the posterior aspect of the heel, which allowed estimation of the moment arms on radiographs. The patients were asked to keep the foot in position and to resist the dorsiflexion moment derived from the external load. Simultaneous exposures were obtained using a calibration plate designed for RSA of the hip (RSA Biomedical). The exposures were done at 10 seconds, 15 seconds, or 3 minutes after the load started, followed by 3 minutes of rest between each load. The patients were asked to rate this test using a VAS score for pain and discomfort to understand whether early loading had altered the perception of loading.

**Table 2:** Protocol for Mechanical Evaluation With Roentgen Stereophotogrammetric Analysis at Weeks 2, 7, 19, and 52^*a*^

	Week After Injury			
	2	7	19	52
Unloaded image	Yes	Yes		
External load 2.5 kg for 15 s^*b*^		Yes	Yes	Yes
External load 10 kg for 10 s		Yes		
External load 10 kg for 3 min		Yes		
External load 15 kg for 15 s^*b*^			Yes	
External load 22.5 kg for 15 s^*b*^				Yes
External load 35 kg for 15 s				Yes

a The external load was put on a pedal that pivoted around an axis. Patients were asked to actively resist the dorsiflexion moment of the pedal before roentgen stereophotogrammetric analysis was conducted.

b The loading session was followed by a 3-minute rest before the next loading session.

Image analyses were performed with investigators blinded to all patient-related information via RSA Biomedical using the UmRSA 4.1 system. The RSA software was used to calculate distances between beads and to compare differences of these measurements between the different time points and loading groups. Tendon force was calculated from the pedal force as previously described.^36^ Percentages of elongation were calculated with correction for the lever arms of the forefoot and the calcaneus bone and are expressed as a percentage per 100 N of tendon force. Lever arms were calculated from lateral computed tomography images, with the center of the talar trochlea as the pivot point (pedal point to trochlear center, trochlear center to center of tendon). These calculations were performed blindly by 1 author (Z.R.). Elastic modulus was calculated as (*Force increase*×*unloaded gap length*) ÷ (*elongation*×*cross-sectional area*). Measurements from the 52-week follow-up were used to calculate the percentage of elongation from the slope of the regression lines for 3 measurements.

### Functional and Patient-Reported Outcomes

The ATRS was used at 19 and 52 weeks to assess patients’ perception of disability. Calf muscle endurance was evaluated using a single-leg standing heel rise test. The test started with the ankle in neutral position followed by heel rises at a tempo of 30 heel rises per minute. The test ended when the patient either stopped or did not reach a minimum height of 5 cm. MuscleLab (Ergotest) computer software and a linear encode sensor attached to the heel were used for evaluation, as previously described.^12^ This test is an adapted protocol from the original studies using a 10° incline.^6,38^ The total number of heel rises and the mean and maximal heel rise heights were recorded, and total muscle work was calculated (body weight × total distance, in joules). Range of motion in the ankle joint was measured in 2 ways: the patient either leaned forward while the knee was bent or held the knee straight while sitting. Calf circumference was measured at the level where the calf muscle was most prominent in order to evaluate muscle atrophy. Ankle circumference was measured approximately 2 cm proximal to the trochlear center to assess swelling around the ankle joint. All functional parameters were normalized to the noninjured side before data analyses.

### Statistical Analysis

The primary outcome variable, tendon elastic modulus at 19 weeks, was analyzed as a continuous variable using Student *t* test. Power analysis was based on a previous study with similar measures by Schepull and Aspenberg,^36^ and it was estimated that a sample of 20 in each group would make it possible to detect a 31% difference in elastic modulus with 80% power. Parameters measured at only 1 time point (eg, heel-rise work) were analyzed using Student *t* test. Structural, mechanical, and functional parameters measured at several time points were analyzed using repeated mixed-effects models (because some data values were missing) followed by the Šidák multiple comparisons test. A spearman correlation test was used for correlation analysis between tendon CSA and muscle function, *p* < 0.05 was considered significant for all tests. We used the GraphPad Prism 9 program for statistical analysis (GraphPad Software).

## Results

Patient background characteristics are given in Table 3.

**Table 3:** Patient Characteristics^*a*^

Variable	Control Group	Loaded Group
Patients, men/women, n	17/3	19/1
Age, y	44.2 ± 7.9	37.3 ± 10.8
Height, cm	179.5 ± 8.2	176.8 ± 6.8
Weight, kg	86.7 ± 9.9	80.2 ± 9.1
Body mass index	27.5 ± 3.7	25.5 ± 3.0
Time from injury to treatment, 0/1/2 d, n	15/4/1	18/2/0
Time from injury to bead insertion, d	13.9 ± 2.2	14.4 ± 2.0
Injured side, left/right, n	10/10	10/10
Injured side, dominant/nondominant, n	9/11	11/9
Activity when injury occurred	Floorball (n = 10), soccer (n = 10), running (n = 4), squash (n = 3), tennis (n = 2), padel (n = 2), volleyball (n = 1), badminton (n = 1), table tennis (n = 1), weight lifting (n = 1), horseback riding (n = 1), circuit training (n = 1), fall (n = 2), at work (n = 1)	

a Values are expressed as mean ± SD unless otherwise noted.

### Tendon Structural and Material Properties

**Figure 4.**
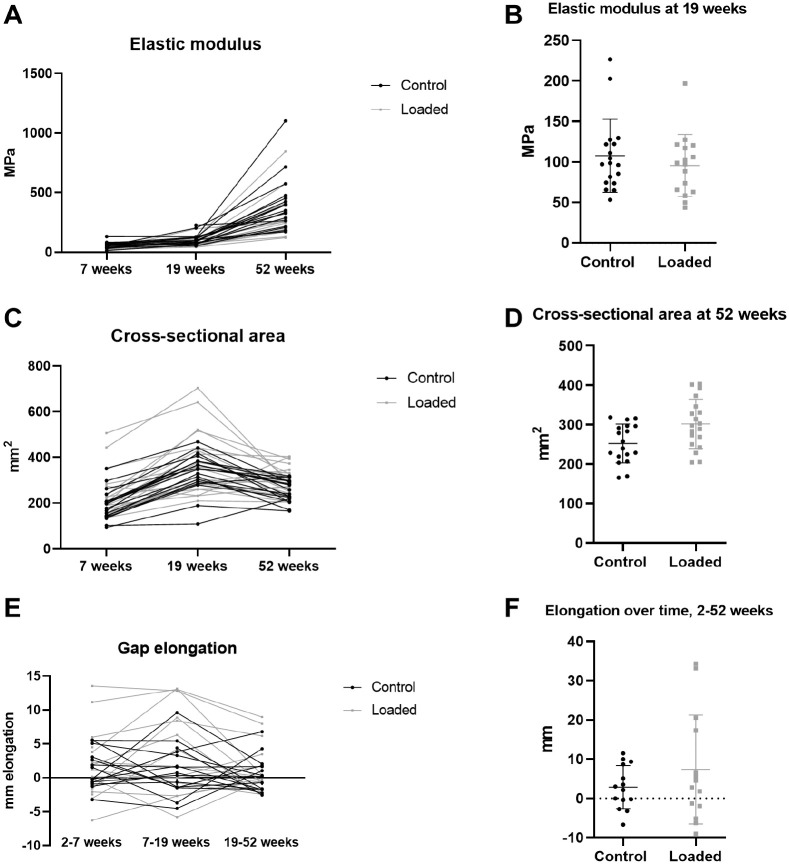
Mechanical data. (A) Tendon elastic modulus at 7, 19, and 52 weeks after injury. (B) Elastic modulus at 19 weeks after injury (primary outcome variable). The lines represent mean values and the bars SDs. (C) Tendon cross-sectional area at 7, 19, and 52 weeks after injury. (D) Tendon cross-sectional area in the 2 groups at 52 weeks after injury. (E) Gap elongation over time (weeks 2-7, weeks 7-19, and weeks 19-52) for each patient. (F) Total gap elongation in the 2 groups between weeks 2 and 52.

The primary outcome variable of this study, tendon elastic modulus at 19 weeks, was a mean ± standard deviation of 95.6 ± 38.2 MPa in the loaded group and 108 ± 45.2 MPa in the control group (95% CI, –41.4 to 17.5 MPa; *P* = .37) (Figure 4). Elastic modulus increased over time but was lower in the loaded group at all time points. We noted an effect of group and time but no interaction (Figure 4; Table 4). Tendon cross-sectional area was also affected by both group and time (*P* = .04; *P* < .001). Mean cross-sectional area was 231 ± 100 mm^2^ in the loaded group versus 188 ± 65 mm^2^ in the control group at 7 weeks and 388 ± 142 mm^2^ versus 335 ± 87 mm^2^, respectively, at 19 weeks. The cross-sectional area at 52 weeks was 20% larger in the loaded group than the control group, 302 ± 62 mm^2^ versus 252 mm^2^± 49 mm^2^, respectively (95% CI, 3.5 to 96 mm^2^). Mean creep at 7 weeks was 1.1% ± 0.8% in the loaded group and 0.84% ± 0.6% in the control group (95% CI, –0.91% to 0.20%). Gap elongation between 2 and 7 weeks in the loaded and control groups was 1.9 ± 5.3 mm versus 1.3 ± 2.8 mm, respectively (95% CI, –3.4 to 4.7 mm), and the overall gap elongation between 2 and 52 weeks was 7.4 ± 14 mm versus 2.9 ± 6 mm, respectively (95% CI, –3.1 to 13 mm) (Figure 4).

**Table 4:** Creep, Tendon Elastic Modulus, Tendon Cross-sectional Area, and Gap Elongation^*a*^

							*P* Values		
	Control Group^*b*^	n	Loaded Group^*b*^	n	Mean Difference	95% CI	Group	Time	Group × Time
Creep at 7 wk, %	0.84 ± 0.61	15	1.14 ± 0.82	13	0.35	−0.91 to 0.20	.20	NA	NA
Elastic modulus, MPa									
7 wk	59.5 ± 27.0^*c*^	15	44.5 ± 20.1^*c*^	15	−15.0	−37.2 to 7.17	.03	<.001	.55
19 wk	108 ± 45.2^*c*^	18	95.6 ± 38.2^*c*^	16	−11.9	−48.0 to 24.1			
52 wk	405 ± 223^*c*^	18	292 ± 180^*c*^	17	−113	−285 to 59.1			
Cross-sectional area, mm^2^									
7 wk	188 ± 65.4	19	231 ± 99.5	20	43.5	−24.0 to 111	.04	<.001	.97
19 wk	335 ± 87.2^*c*^	18	388 ± 142^*c*^	17	52.3	−49.9 to 155			
52 wk	252 ± 49.2^*c*^	18	302 ± 62.4^*d*^	19	49.7	3.46 to 96.0			
Gap elongation (distance between beads), mm									
2-7 wk	1.31 ± 2.76	14	1.94 ± 5.30	15	0.63	−3.40 to 4.66	.26	.13	.37
7-19 wk	1.19 ± 3.71	15	4.05 ± 6.41	14	2.85	−2.25 to 7.96			
19-52 wk	0.40 ± 2.63	15	1.43 ± 3.80	14	1.03	−2.11 to 4.17			
2-52 wk	2.86 ± 5.52	14	7.35 ± 13.9	14	4.49	−3.07 to 12.7	.27	NA	NA
7-52 wk	1.59 ± 4.55	15	5.47 ± 8.71	15	3.87	−1.32 to 9.07	.14	NA	NA

a Data were analyzed using repeated mixed-effects models with group and time as main factors followed by the Šidák multiple comparisons test, except for creep and gap elongation (2-52 weeks and 7-52 weeks), which were analyzed using independent Student *t* test. NA, not analyzed because the time factor was not available.

b Values are expressed as mean ± SD.

c Significant effect of time compared with the earlier time point.

d Significant effect of group.

### Functional and Patient-Reported Outcomes

**Figure 5.**
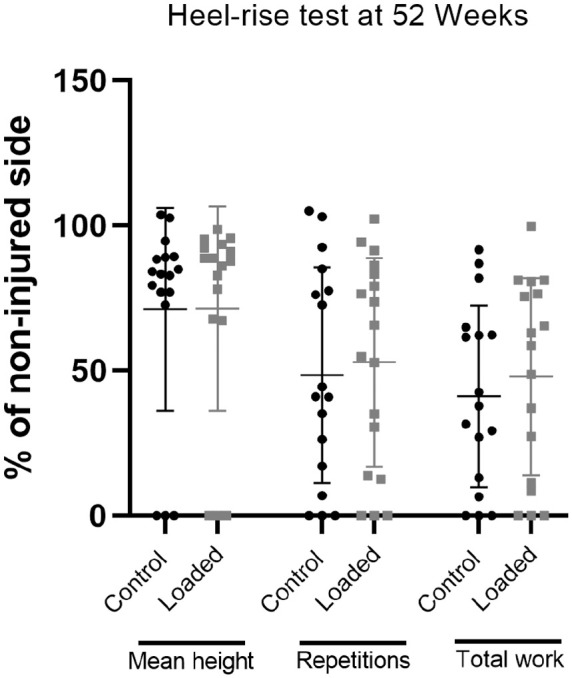
Results from a heel-rise test at 52 weeks after injury presented as percentage of the noninjured side: the mean height of all repetitions, the total number of repetitions, and total heel-rise work. The lines represent mean values and the bars SDs.

The ATRS increased between 19 and 52 weeks (*P* < .001). The mean score was 50 ± 19 at 19 weeks and 83 ± 18 at 52 weeks in the loaded group compared with 59 ± 15 and 76 ± 16, respectively, in the control group (Table 5). We found no effect of group (*P* = .87) but did note an interaction between group and time (*P* = .008). Maximal heel-rise height increased with time in the loaded and control groups from a mean limb symmetry index of 42% ± 27% and 44% ± 29%, respectively, of the noninjured side at 19 weeks to 69% ± 34% and 67% ± 29%, respectively, at 52 weeks (*P* < .001) (Table 6). There was no group effect or interaction. We found a weak correlation with tendon cross-sectional area at 19 weeks and the maximal heel-rise height at 52 weeks (Spearman correlation *r* = 0.357; *P* = .04). The total heel-rise work at 52 weeks was 48% ± 34% of the noninjured leg in the loaded group versus 41% ± 31% in the control group (*P* = .55) (Figure 5). Calf circumference was 96% of the noninjured side in both groups at both time points (95% CI, –1.4% to 2.5% and −0.9% to 2.7% at 19 and 52 weeks, respectively). The range of motion in the ankle joint was 86% ± 15% of the noninjured side at 19 weeks and 95% ± 15% at 52 weeks in the loaded group compared with 90% ± 9% and 94% ± 9% at these time points in the control group. The VAS at 7 weeks showed a mean score of 1.6 ± 1.8 versus 2.8 ± 2.2 in the respective groups (95% CI, –2.7 to 0.2; *P* = .1).

**Table 5:** Patient-Reported Outcomes for ATRS, Calf Circumference, Ankle Circumference, Ankle ROM, and Maximal Heel-Rise Height^*a*^

	19 Weeks				52 Weeks				*P* Values		
	Control^*b*^ (n = 18)	Loaded^*b*^ (n = 19)	Mean Difference	95% CI	Control^*b*^ (n = 18)	Loaded^*b*^ (n = 18)	Mean Difference	95% CI	Group	Time	Group × Time
ATRS	58.9 ± 15.4	50.4 ± 19.2	−8.6	−21 to 4.3	76.2 ± 15.4	83.2 ± 18.0	7.0	−5.9 to 20	.87	<.001	.008
Calf circumference, %	95.8 ± 2.7	96.3 ± 2.5	0.6	−1.4 to 2.5	95.6 ± 2.3	96.6 ± 2.7	1.1	−0.9 to 3.0	.28	.89	.54
Ankle circumference, %	103 ± 3.1	105 ± 3.3	1.6	−0.6 to 3.7	101 ± 2.3	102 ± 2.3	0.5	−1.5 to 2.7	.20	<.001	.27
Ankle ROM: straight knee, %	96.7 ± 11.4	95.4 ± 15.5	−1.3	−11 to 8.1	95.9 ± 11.2	99.4 ± 11.2	3.5	−5.9 to 13	.74	.52	.34
Ankle ROM: bent knee, %	89.3 ± 9.4	85.9 ± 14.5^*c*^	−3.4	−13 to 6.0	94.3 ± 9.1^*c*^	95.2 ± 15.0^*c*^	0.9	−8.6 to 10	.73	.002	.30
Maximal heel-rise height, %	43.9 ± 28.8	41.7 ± 27.3	−2.2	−25 to 20	67.4 ± 29.5	68.7 ± 33.9	2.0	−21 to 25	.99	<.001	.71

a Data were analyzed with repeated mixed-effects models with group and time as main factors followed by the Šidák multiple comparisons test. Creep and total gap elongation were analyzed with an independent Student *t* test. Values for circumference, ROM, and heel-rise height are the limb symmetry index (percentage of the noninjured side). ATRS, Achilles tendon Total Rupture Score; ROM, range of motion.

b Values are expressed as mean ± SD.

c n = 18.

**Table 6:** Calf Circumference, Ankle Circumference, Ankle ROM, and Heel-Rise Test Data at 19 and 52 Weeks Presented as Absolute Numbers^*a*^

	Control		Loading	
	Injured	Intact	Injured	Intact
19 wk				
Calf circumference, cm	36.3 ± 1.7	38.0 ± 2.2	36.8 ± 2.5	38.2 ± 2.4
Ankle circumference, cm	22.9 ± 1.1	22.2 ± 0.9	23.8 ± 1.4	22.7 ± 1.3
Ankle ROM: straight knee, deg	55.7 ± 9.5	57.7 ± 8.2	57.3 ± 9.4	60.5 ± 7.8
Ankle ROM: bent knee, deg	29.3 ± 4.3	32.9 ± 4.5	28.5 ± 5.0	33.4 ± 4.4
Maximal heel-rise height, cm	4.7 ± 3.1	10.3 ± 1.5	4.7 ± 3.1	12.2 ± 2.0
52 wk				
Calf circumference, cm	36.6 ± 2.1	38.3 ± 2.3	37.0 ± 3.0	38.3 ± 2.7
Ankle circumference, cm	22.5 ± 0.9	22.2 ± 0.9	22.9 ± 1.4	22.4 ± 1.5
Ankle ROM: straight knee, deg	55.3 ± 10.5	57.7 ± 8.3	58.3 ± 16.1	61.4 ± 8.6
Ankle ROM: bent knee, deg	30.6 ± 5.1	32.6 ± 4.6	32.8 ± 4.9	34.8 ± 6.0
Maximal heel-rise height, cm	7.1 ± 3.3	10.6 ± 2.0	9.0 ± 4.3	12.5 ± 1.6
Mean heel-rise height, cm	6.5 ± 2.8	8.8 ± 1.6	7.7 ± 3.8	9.9 ± 1.8
No. of heel rises	17.8 ± 12.7	45.1 ± 46.7	23.5 ± 15.9	39.8 ± 11.9
Total heel-rise height, cm	140.3 ± 103.8	402.7 ± 426.3	219.7 ± 160.4	386.2 ± 132.4
Total work, J	1097 ± 888	3045 ± 3254	1576 ± 1355	2957 ± 1229

a Values are expressed as mean ± SD. ROM, range of motion.

### Return to Work and Sports

Mean time away from work was 55.7 ± 88.2 days in the loaded group compared with 55.3 ± 65 days in the control group (*P* = .99). Some level of sports-related activity was regained in 15 and 13 patients in the loaded and control groups, respectively with a mean of 239 and 188 days, respectively (*P* = .1). A total of 4 patients had not returned to any sports, and 8 patients did not respond (including the two patients with complete rerupture). Further, 11 (28%) of the 40 patients had returned to their preinjury level of sports, whereas 21 (53%) patients reported that they had not reached this level at 52 weeks.

### Compliance and Complications

Most patients followed the instructed loading protocol without any reduction in repetitions or load (Table 7). Two patients were missing in the 19-week mechanical testing, 1 because of pregnancy and 1 because of traveling. One patient was excluded from the study because of inability to adhere to the study protocol. A further 2 patients were excluded from the mechanical analyses because of incorrect bead placement outside of the tendon. A total of 3 patients had reruptures, of which 2 were complete and 1 was incomplete. The incomplete rerupture occurred during heel-rise exercises at 18 weeks after injury (loaded group); this patient backed in time 4 weeks in the rehabilitation protocol. One complete rerupture occurred during a sudden step at 8 weeks after injury (control group) and was treated with an ankle orthosis for 6 weeks. Another complete rerupture occurred while the patient was performing heel-rise exercises at approximately 10 weeks after injury (loaded group); this patient was treated with a plaster cast in plantarflexion for 2 weeks followed by an ankle orthosis for 5 weeks. The patient with the incomplete rerupture was followed and included as planned. A total of 3 patients (1 in the loaded group) had confirmed deep vein thrombosis. The patients were treated with tinzaparin (low-molecular-weight heparin) or rivaroxaban, and they continued in the study.

**Table 7:** Number of Patients Who Reported <20 Repetitions at Each Training Session With the Training Pedal Used in the Early Loading Group

	Day 1	Day 2	Day 3	Day 4	Day 5	Day 6	Day 7
Week 3 (am/pm)	5/4	3/3	3/2	2/1	1/2	1/1	1/1
Week 4 (am/pm)	1/2	0/0	0/0	0/2	1/0	0/1	1/1
Week 5 (am/pm)	2/1	2/2	0/0	0/1	0/1	1/0	0/2
Week 6 (am/pm)	1/1	0/0	0/0	0/2	0/0	0/0	0/1
Week 7 (am/pm)	2/2	1/2	2/2	2/1	1/1	0/2	0/0

## Discussion

In contrast to our hypothesis, early tensile loading had a negative effect on elastic modulus but resulted in an increased cross-sectional area of the tendon at 52 weeks after injury. Patient-reported outcome, ankle circumference, range of motion of the ankle joint, and maximal heel-rise height improved over time in both groups, with no additive or diminishing effect of early tensile loading. At 52 weeks after injury, there was a 32% reduction in maximal heel-rise height of the noninjured side in the loaded group and a 33% reduction in the control group, and the total heel-rise work was <50%. We found no increased risk for adverse events after early tensile loading measured by complete reruptures, deep vein thrombosis, and tendon gap length.

Early tensile loading has been shown to have beneficial effects on tendon material properties in animal models and surgically treated tendons.^1,13,36^ The same tensile loading protocol appears to have a different effect if applied after surgical repair or nonsurgical treatment. One study found that surgically treated tendons had improved elastic modulus and no difference in cross-sectional area compared with controls.^36^ A possible explanation for this finding might be that the surgical repair affects the biological response of the tensile loading regimen by inhibiting excessive loading in the healing tissue. A moderate loading regimen in the rat tendon, for example, favors a biological response through the mechanism of mechanotransduction, whereas heavy loading also induces tissue damages.^19^ Furthermore, heavy loading extends the proinflammatory response, which might delay remodeling of the tendon.^18^ Small tissue damage induced via dry needling is comparable with damage related to heavy loading in rats, and such needle damage induces a gene expression response similar to that of heavy loading, thus leading to improved strength.^17^ However, this improved strength can be explained by the increased cross-sectional area alone, not a better overall tissue quality.^17^ In the present study, however, tendon cross-sectional area at 19 weeks showed a weak correlation with heel-rise height at 52 weeks, which indicates a functional effect of tendon size. A correlation between tendon thickness and muscle force recovery is in line with a previous suggestion that early structural measurements could be used to predict later functional results.^46^

*Early functional rehabilitation* is an umbrella term that covers a wide range of different exercise protocols. Some protocols include specific tensile loading exercises, but, most commonly, functional rehabilitation includes early weightbearing in an ankle orthosis and unloaded motion exercises.^45^ This type of rehabilitation is widely accepted as preferable compared with immobilization, although the reported outcomes diverge. Some studies have shown improved results in return to sports,^8,34^ shorter rehabilitation time,^28^ smaller deficit in heel-rise height,^34^ and better isokinetic strength.^26^ Conversely, other studies have presented no improved outcome.^4,9,12,22^ Both groups in the current study were treated with an accelerated rehabilitation program containing early weightbearing and unloaded range of motion exercises. The only difference between the 2 groups was the tensile loading exercises twice daily between weeks 3 and 7. We found no difference in the ATRS at 19 or 52 weeks after injury. This is similar to findings in other studies of both surgically and nonsurgically treated tendons^3,4,12,34^; hence, early accelerated rehabilitation appears to have little effect on the results perceived by the patients. The ATRS at 52 weeks in our study is comparable with the score in surgically treated patients with the same loading protocol.^36^ However, the number of reruptures was higher after nonsurgical treatment (n = 3; 7.5%) compared with surgical treatment (n = 1; 2.5%).

We found no statistical difference in return to work or sports, which contrasts with earlier studies that compared immobilization and accelerated rehabilitation.^8,28,34^ However, for return to any type of sports, we found a numerical difference of 51 days between the 2 groups. This can be of clinical relevance and of great psychological value. Furthermore, we found that 70% of the patients had returned to sports but only 28% reported that they had returned to the same level. This is slightly higher compared with a previous study on surgically treated patients by Eliasson et al^12^ and similar to a meta-analysis by Zellers et al.^44^ That meta-analysis showed a mean of 6 months for return to play. According to our findings, there appears to be no additive effect of early tensile loading on top of the accelerated rehabilitation regimen for these outcomes. However, there is also no apparent, increased risk of major complications such as reruptures or deep vein thrombosis. A recent meta-analysis found that early functional rehabilitation in nonsurgically treated tendons was comparable with open surgical treatment with early immobilization.^43^ That meta-analysis also showed an imbalance in rehabilitation studies in surgically or nonsurgically treated groups. Of the 29 analyzed studies, only 2 entailed multiple, nonsurgically treated groups. More studies with nonsurgical treatment are needed because a comparison with surgical treatment appears unfeasible.

Muscle atrophy, defined as deficiencies in heel-rise height, total muscle work, and side-to-side variations in calf circumference, was substantial in both groups at 52 weeks after injury. Although heel-rise height improved between weeks 19 and 52, there was no additional improvement with early tensile loading. Long-lasting muscle atrophy is a known consequence of Achilles tendon ruptures.^3,4,20,21,32^ Our patients performed 48 and 41% of total muscle work of the noninjured side, in each respective group. The muscle atrophy was also reflected in a smaller calf circumference. This is somewhat surprising because patients undergoing surgical treatment typically reach 70% of the noninjured side.^7,12,30,32,41^ The heel-rise height at 52 weeks differs in our study compared with the study by Schepull et al,^37^ who used the same loading regimen after surgical repair. The heel-rise height was approximately 68% of the intact side in our study compared with 79% in the Schepull et al study.^37^ The observed differences are likely related to the nonsurgical treatment protocol, which can result in more severe muscle atrophy of the soleus muscle compared with surgical treatment.^20^ Such a large difference is concerning because heel-rise performance 1 year after an Achilles tendon rupture is associated with long-term alterations in ankle biomechanics.^5^

Similar to the previous study on surgically treated Achilles tendon ruptures, we found no difference in gap elongation between the 2 groups in this study.^36^ However, the early loading group had a mean elongation that was 4.5 mm more than that of the control group, and this could be of clinical relevance—for example, in jumping activities. The Achilles tendon often elongates during tendon healing, and extensive tendon elongation is associated with poor patient outcome.^25,40^ However, the effect of less pronounced tendon elongation on patient recovery is controversial possibly due to a variety of different measurement techniques such as magnetic resonance imaging, RSA, Achilles tendon resting angle, or heel-rise height.^10,11,32,35,36^ We measured gap elongation between the beads inserted 2 weeks after the injury and did not measure total tendon length. Okoroha et al^31^ showed parallels between total tendon elongation and gap elongation. However, we lacked measurements on tendon elongation before the 2-week follow-up, and a substantial amount of total tendon elongation could have occurred before bead insertion.^29,31^

This study had some limitations. In the measurements of the material properties, a number of simplifying assumptions had to be made, such as tissue homogeneity, a consistent cross-sectional area along the tendon between the beads, and a linear load deformation relationship. However, these simplifications were done for both groups and should therefore not influence group differences. Additionally, we did not perform double RSA examinations to assess measurement error, because this has been done previously by our group using the same instruments.^37^ The heel-rise test in this study entailed an adapted protocol that differed slightly from the setup, as we used a neutral starting position compared with the original studies^6,38^ that used a 10° incline in the starting position. This could lead to different findings if the tendon is elongated.

In conclusion, early tensile loading in nonsurgically treated Achilles tendon ruptures resulted in an increased tendon thickness compared with unloaded motion exercises and early weightbearing in an ankle orthosis. These early tensile loading exercises were well tolerated and appreciated by the patients; however, the material in the healing tendon was less stiff after this regimen, and the muscle atrophy was still pronounced compared with that of the noninjured side. We observed no apparent increased risk of major complications, such as reruptures or deep vein thrombosis. Our findings indicate the importance of high-quality scientific evaluation even when minor changes are implemented in health care routines.

## References

[1] Andersson T Eliasson P Hammerman M Sandberg O Aspenberg P . Low-level mechanical stimulation is sufficient to improve tendon healing in rats. J Appl Physiol (1985). 2012;113(9):1398-1402. 22936727 10.1152/japplphysiol.00491.2012

[2] Aspenberg P Schepull T . Substantial creep in healing human Achilles tendons: a pilot study. Muscles Ligaments Tendons J. 2015;5(3):151-155. 26605187 10.11138/mltj/2015.5.3.151 PMC4617213

[3] Barfod KW Bencke J Lauridsen HB Ban I Ebskov L Troelsen A . Nonoperative dynamic treatment of acute Achilles tendon rupture: the influence of early weight-bearing on clinical outcome: a blinded, randomized controlled trial. J Bone Joint Surg Am. 2014;96(18):1497-1503. 25232073 10.2106/JBJS.M.01273

[4] Barfod KW Hansen MS Hölmich P Kristensen MT Troelsen A . Efficacy of early controlled motion of the ankle compared with immobilisation in non-operative treatment of patients with an acute Achilles tendon rupture: an assessor-blinded, randomised controlled trial. Br J Sports Med. 2020;54(12):719-724. 31597624 10.1136/bjsports-2019-100709

[5] Brorsson A Willy RW Tranberg R Gravare Silbernagel K . Heel-rise height deficit 1 year after Achilles tendon rupture relates to changes in ankle biomechanics 6 years after injury. Am J Sports Med. 2017;45(13):3060-3068. 28783473 10.1177/0363546517717698

[6] Byrne C Keene DJ Lamb SE Willett K . Intrarater reliability and agreement of linear encoder derived heel-rise endurance test outcome measures in healthy adults. J Electromyogr Kinesiol. 2017;36:34-39. 28719820 10.1016/j.jelekin.2017.07.004

[7] Carmont MR Zellers JA Brorsson A Silbernagel KG Karlsson J Nilsson-Helander K . No difference in strength and clinical outcome between early and late repair after Achilles tendon rupture. Knee Surg Sports Traumatol Arthrosc. 2020;28(5):1587-1594. 30594954 10.1007/s00167-018-5340-5 PMC7176605

[8] Cetti R Henriksen LO Jacobsen KS . A new treatment of ruptured Achilles tendons: a prospective randomized study. Clin Orthop Relat Res. 1994;308:155-165. 7955677

[9] Coopmans L Amaya Aliaga J Metsemakers WJ , et al. Accelerated rehabilitation in nonoperative management of acute Achilles tendon ruptures—a systematic review and meta-analysis. J Foot Ankle Surg. 2022;61(1):157-162. 34400090 10.1053/j.jfas.2021.07.007

[10] Cramer A Rahdi E Hansen MS Sandholdt H Hölmich P Barfod KW . No clinically relevant difference between operative and non-operative treatment in tendon elongation measured with the Achilles tendon resting angle (ATRA) 1 year after acute Achilles tendon rupture. Knee Surg Sports Traumatol Arthrosc. 2021;29(5):1617-1626. 33386883 10.1007/s00167-020-06391-w

[11] Diniz P Pacheco J Guerra-Pinto F Pereira H Ferreira FC Kerkhoffs G . Achilles tendon elongation after acute rupture: is it a problem? A systematic review. Knee Surg Sports Traumatol Arthrosc. 2020;28(12):4011-4030. 32363475 10.1007/s00167-020-06010-8

[12] Eliasson P Agergaard AS Couppe C , et al. The ruptured Achilles tendon elongates for 6 months after surgical repair regardless of early or late weightbearing in combination with ankle mobilization: a randomized clinical trial. Am J Sports Med. 2018;46(10):2492-2502. 29965789 10.1177/0363546518781826

[13] Eliasson P Andersson T Aspenberg P . Achilles tendon healing in rats is improved by intermittent mechanical loading during the inflammatory phase. J Orthop Res. 2012;30(2):274-279. 21809382 10.1002/jor.21511

[14] Ganestam A Kallemose T Troelsen A Barfod KW . Increasing incidence of acute Achilles tendon rupture and a noticeable decline in surgical treatment from 1994 to 2013: a nationwide registry study of 33,160 patients. Knee Surg Sports Traumatol Arthrosc. 2016;24(12):3730-3737. 25697284 10.1007/s00167-015-3544-5

[15] Gould HP Bano JM Akman JL Fillar AL . Postoperative rehabilitation following Achilles tendon repair: a systematic review. Sports Med Arthrosc Rev. 2021;29(2):130-145. 33972490 10.1097/JSA.0000000000000309

[16] Haapasalo H Peltoniemi U Laine HJ Kannus P Mattila VM . Treatment of acute Achilles tendon rupture with a standardised protocol. Arch Orthop Trauma Surg. 2018;138(8):1089-1096. 29725765 10.1007/s00402-018-2940-y

[17] Hammerman M Aspenberg P Eliasson P . Microtrauma stimulates rat Achilles tendon healing via an early gene expression pattern similar to mechanical loading. J Appl Physiol (1985). 2014;116(1):54-60. 24177691 10.1152/japplphysiol.00741.2013

[18] Hammerman M Blomgran P Dansac A Eliasson P Aspenberg P . Different gene response to mechanical loading during early and late phases of rat Achilles tendon healing. J Appl Physiol (1985). 2017;123(4):800-815. 28705996 10.1152/japplphysiol.00323.2017

[19] Hammerman M Dietrich-Zagonel F Blomgran P Eliasson P Aspenberg P . Different mechanisms activated by mild versus strong loading in rat Achilles tendon healing. PLoS One. 2018;13(7):e0201211. 10.1371/journal.pone.0201211 PMC6059492 30044869

[20] Heikkinen J Lantto I Flinkkila T , et al. Soleus atrophy is common after the nonsurgical treatment of acute Achilles tendon ruptures: a randomized clinical trial comparing surgical and nonsurgical functional treatments. Am J Sports Med. 2017;45(6):1395-1404. 28282504 10.1177/0363546517694610

[21] Heikkinen J Lantto I Piilonen J , et al. Tendon length, calf muscle atrophy, and strength deficit after acute Achilles tendon rupture: long-term follow-up of patients in a previous study. J Bone Joint Surg Am. 2017;99(18):1509-1515. 28926379 10.2106/JBJS.16.01491

[22] Huang J Wang C Ma X Wang X Zhang C Chen L . Rehabilitation regimen after surgical treatment of acute Achilles tendon ruptures: a systematic review with meta-analysis. Am J Sports Med. 2015;43(4):1008-1016. 24793572 10.1177/0363546514531014

[23] Hutchison AM Topliss C Beard D Evans RM Williams P . The treatment of a rupture of the Achilles tendon using a dedicated management programme. Bone Joint J. 2015;97(4):510-515. 25820890 10.1302/0301-620X.97B4.35314

[24] Huttunen TT Kannus P Rolf C Fellander-Tsai L Mattila VM . Acute Achilles tendon ruptures: incidence of injury and surgery in Sweden between 2001 and 2012. Am J Sports Med. 2014;42(10):2419-2423. 25056989 10.1177/0363546514540599

[25] Kangas J Pajala A Ohtonen P Leppilahti J . Achilles tendon elongation after rupture repair: a randomized comparison of 2 postoperative regimens. Am J Sports Med. 2007;35(1):59-64. 16973901 10.1177/0363546506293255

[26] Kangas J Pajala A Siira P Hamalainen M Leppilahti J . Early functional treatment versus early immobilization in tension of the musculotendinous unit after Achilles rupture repair: a prospective, randomized, clinical study. J Trauma. 2003;54(6):1171-1180. 12813340 10.1097/01.TA.0000047945.20863.A2

[27] Manent A Lopez L Corominas H , et al. Acute Achilles tendon ruptures: efficacy of conservative and surgical (percutaneous, open) treatment-a randomized, controlled, clinical trial. J Foot Ankle Surg. 2019;58(6):1229-1234. 31679677 10.1053/j.jfas.2019.02.002

[28] Mortensen HM Skov O Jensen PE . Early motion of the ankle after operative treatment of a rupture of the Achilles tendon: a prospective, randomized clinical and radiographic study. J Bone Joint Surg Am. 1999;81(7):983-990. 10428130 10.2106/00004623-199907000-00011

[29] Mortensen NH Saether J Steinke MS Staehr H Mikkelsen SS . Separation of tendon ends after Achilles tendon repair: a prospective, randomized, multicenter study. Orthopedics. 1992;15(8):899-903. 1508765 10.3928/0147-7447-19920801-06

[30] Nilsson-Helander K Silbernagel KG Thomeé R , et al. Acute Achilles tendon rupture: a randomized, controlled study comparing surgical and nonsurgical treatments using validated outcome measures. Am J Sports Med. 2010;38(11):2186-2193. 20802094 10.1177/0363546510376052

[31] Okoroha KR Ussef N Jildeh TR , et al. Comparison of tendon lengthening with traditional versus accelerated rehabilitation after Achilles tendon repair: a prospective randomized controlled trial. Am J Sports Med. 2020;48(7):1720-1726. 32203675 10.1177/0363546520909389

[32] Olsson N Silbernagel KG Eriksson BI , et al. Stable surgical repair with accelerated rehabilitation versus nonsurgical treatment for acute Achilles tendon ruptures: a randomized controlled study. Am J Sports Med. 2013;41(12):2867-2876. 24013347 10.1177/0363546513503282

[33] Perren SM . Evolution of the internal fixation of long bone fractures: the scientific basis of biological internal fixation. Choosing a new balance between stability and biology. J Bone Joint Surg Br. 2002;84(8):1093-1110. 12463652 10.1302/0301-620x.84b8.13752

[34] Porter MD Shadbolt B . Randomized controlled trial of accelerated rehabilitation versus standard protocol following surgical repair of ruptured Achilles tendon. ANZ J Surg. 2015;85(5):373-377. 25366811 10.1111/ans.12910

[35] Rosso C Vavken P Polzer C , et al. Long-term outcomes of muscle volume and Achilles tendon length after Achilles tendon ruptures. Knee Surg Sports Traumatol Arthrosc. 2013;21(6):1369-1377. 23370984 10.1007/s00167-013-2407-1

[36] Schepull T Aspenberg P . Early controlled tension improves the material properties of healing human Achilles tendons after ruptures: a randomized trial. Am J Sports Med. 2013;41(11):2550-2557. 24005873 10.1177/0363546513501785

[37] Schepull T Kvist J Andersson C Aspenberg P . Mechanical properties during healing of Achilles tendon ruptures to predict final outcome: a pilot Roentgen stereophotogrammetric analysis in 10 patients. BMC Musculoskelet Disord. 2007;8:116. 18039357 10.1186/1471-2474-8-116 PMC2244624

[38] Silbernagel KG Nilsson-Helander K Thomeé R Eriksson BI Karlsson J . A new measurement of heel-rise endurance with the ability to detect functional deficits in patients with Achilles tendon rupture. Knee Surg Sports Traumatol Arthrosc. 2010;18(2):258-264. 19690833 10.1007/s00167-009-0889-7

[39] Soroceanu A Sidhwa F Aarabi S Kaufman A Glazebrook M . Surgical versus nonsurgical treatment of acute Achilles tendon rupture: a meta-analysis of randomized trials. J Bone Joint Surg Am. 2012;94(23):2136-2143. 23224384 10.2106/JBJS.K.00917 PMC3509775

[40] Svensson RB Couppé C Agergaard A-S , et al. Persistent functional loss following ruptured Achilles tendon is associated with reduced gastrocnemius muscle fascicle length, elongated gastrocnemius and soleus tendon, and reduced muscle cross-sectional area. Transl Sports Med. 2019;2(6):316-324.

[41] Valkering KP Aufwerber S Ranuccio F Lunini E Edman G Ackermann PW . Functional weight-bearing mobilization after Achilles tendon rupture enhances early healing response: a single-blinded randomized controlled trial. Knee Surg Sports Traumatol Arthrosc. 2017;25(6):1807-1816. 27539402 10.1007/s00167-016-4270-3 PMC5487693

[42] Valstar ER Gill R Ryd L Flivik G Borlin N Karrholm J . Guidelines for standardization of radiostereometry (RSA) of implants. Acta Orthop. 2005;76(4):563-572. 16195075 10.1080/17453670510041574

[43] Wu Y Mu Y Yin L Wang Z Liu W Wan H . Complications in the management of acute Achilles tendon rupture: a systematic review and network meta-analysis of 2060 patients. Am J Sports Med. 2019;47(9):2251-2260. 30781966 10.1177/0363546518824601

[44] Zellers JA Carmont MR Gravare Silbernagel K . Return to play post-Achilles tendon rupture: a systematic review and meta-analysis of rate and measures of return to play. Br J Sports Med. 2016;50(21):1325-1332. 27259751 10.1136/bjsports-2016-096106 PMC5136353

[45] Zellers JA Christensen M Kjaer IL Rathleff MS Silbernagel KG . Defining components of early functional rehabilitation for acute Achilles tendon rupture: a systematic review. Orthop J Sports Med. 2019;7(11):2325967119884071. 10.1177/2325967119884071 PMC6878623 31803789

[46] Zellers JA Cortes DH Pohlig RT Silbernagel KG . Tendon morphology and mechanical properties assessed by ultrasound show change early in recovery and potential prognostic ability for 6-month outcomes. Knee Surg Sports Traumatol Arthrosc. 2019;27(9):2831-2839. 30415387 10.1007/s00167-018-5277-8 PMC6510650

